# The Investigation of Metabonomic Pathways of Serum of Iranian Women with Recurrent Miscarriage Using ^1^H NMR

**DOI:** 10.1155/2021/3422138

**Published:** 2021-11-03

**Authors:** Mahbobeh Latifimehr, Ali Asghar Rastegari, Zahra Zamani, Pezhman Fard Esfahani, Leila Nazari

**Affiliations:** ^1^Department of Molecular and Cell Biochemistry, Falavarjan Branch, Islamic Azad University, Isfahan, Iran; ^2^Department of Biochemistry, Pasteur Institute of Iran, Tehran, Iran; ^3^Department of Obstetrics and Gynecology Preventative Gynecology Research Center, Shahid Beheshti University of Medical Sciences, Tehran, Iran

## Abstract

**Purpose:**

Recurrent miscarriage applies to pregnancy loss expulsion of the fetus within the first 24 weeks of pregnancy. This study is aimed at comparatively investigating the sera of women with RM with those who have no record of miscarriages to identify if there were any metabolite and metabolic pathway differences using ^1^H NMR spectroscopy.

**Methods:**

Serum samples were collected from women with RM (*n* = 30) and those who had no records of RM (*n* = 30) to obtain metabolomics information. ^1^H NMR spectroscopy was carried out on the samples using Carr Purcell Meiboom Gill spin echo; also, Partial Least Squares Discriminant Analysis was performed in MATLAB software using the ProMetab program to obtain the classifying chemical shifts; the metabolites were identified by using the Human Metabolome Database (HMDB) in both the experimental and control groups. The pathway analysis option of the *Metaboanalyst.ca* website was used to identify the changed metabolic pathways.

**Results:**

The results of the study revealed that 14 metabolites were different in the patients with RM. Moreover, the pathway analysis showed that taurine and hypotaurine metabolism along with phenylalanine, tyrosine, and tryptophan biosynthesis was significantly different in patients with RM.

**Conclusion:**

The present study proposes that any alteration in the above metabolic pathways might lead to metabolic dysfunctions which may result in a higher probability of RM.

## 1. Introduction

Recurrent miscarriage (RM) refers to cases where three or more recurrent miscarriages occur before the twentieth week of pregnancy [1]. According to the World Health Organization (WHO), RM refers to the expulsion or death of a fetus weighing less or more than 500 grams within the 20^th^ to 24^th^ week of pregnancy [2]. Moreover, other terms like abortion, habitual abortion, and spontaneous abortion are interchangeably used to describe RM [3]. However, in 50% of the cases, the cause of abortion is unknown [4] involving repeated participation of various factors such as chromosomal, placental, genetic, anatomic, endocrine, infectious, environmental, and immunologic abnormalities [5]. RM may be described as primary which refers to cases that have not experienced any live birth and secondary RM denoting patients with at least one live child [6]. Previously conducted studies demonstrated that women who experienced miscarriages are prone to having a higher risk of miscarriage in their subsequent pregnancy than those who had successful deliveries [7]. Severe problems affecting people with RM are psychological disorders and sometimes even marital decline [8].

Metabonomics can be used to identify the metabolic patterns in various types of diseases [9]. In addition to providing biochemical information on cells and tissues, metabonomic data can identify unknown genes [10]. Analysis of low molecular weight (LMW) compounds in biological fluids can be used to screen, diagnose congenital metabolic errors [11] and evaluate fetal disorders [12]. Liquid chromatography along with mass spectrometry (LC-MS), nuclear magnetic resonance (NMR), and gas chromatography MS (GC-MS) as some techniques is implemented to identify and quantify metabolites [13]. Metabolites such as plasma, urine, milk, saliva, amniotic fluid, tissue extracts, cerebrospinal fluid, bile, fecal extracts and semen can be detected by the above techniques [14–21].

^1^H NMR though a less-sensitive technique than LC-MS is nondestructive and more economical, and no sample preparation is needed even in case of tissues. The resonance profiles from NMR spectra are based on the chemical structure of molecules and can be used to detect diseases [22]. Metabolites act as linkers to the information-rich genome and functional phenotype and also present products of the cells' regulatory processes [23]. The information density of NMR spectrum is very high, and the data is typically analyzed by multivariate statistical analysis. In biofluid analysis, the signals of protein and other biological macromolecules usually mask the identification of low-level molecular weight metabolites [24]. It is through the Carr Purcell Meiboom Gill (CPMG) method that LMWs can be identified. Targeted and nontargeted methods are two distinct approaches to processing NMR spectra [25]. In both methods, different multivariate statistical methods such as Principal Component Analysis (PCA) and Partial Least Squares Discriminant Analysis (PLS-DA) are used to search for significant differences between the spectra. Another noteworthy point about NMR spectra analysis is the variation that exists because of peak position and line width that is controlled by data reduction [24]. The differentiating metabolites in different groups are identified by their chemical shifts using the Human Metabolome Database (http://www.hmdb.ca) [25], and the metabolic pathways are detected by MetaboAnalyst Database Website (http://www.metaboanalyst.ca) [26]. Since approximately 50% of recurrent miscarriages occur because of some unknown reasons, understanding the alteration of cell metabolism in women with RM is essential.

Earlier workers have shown the use of serum metabonomics with ^1^H NMR in women with idiopathic recurrent spontaneous miscarriage during the implantation window [27] and identified some amino acids associated with it. Efforts have been made on transcriptomic studies to differentiate women with recurrent miscarriages and repeated implantation failures and fertile women to no avail [28].

Our study investigated the difference in metabonomics of sera of patients with RM with those women who had no history of miscarriages and had at least two children. The aim of this study was to investigate the metabolic pathways of recurrent miscarriage and to identify differentiating metabolites which may lead to possible biomarkers in further studies.

## 2. Materials and Methods

### 2.1. Subject Collection

Women with RM who were referred to Taleghani Hospital in Tehran to receive medical treatment were selected as participants of the study. At first, the study was approved by the Ethics Committee (IR.IAU.NAJAFABAD.REC.1397.020) to meet the ethical requirements. Then, the researchers endeavored to obtain informed written consent from the participants of the investigation. The study included two groups of (1) women with a history of RM and (2) women who had at least two children and no history of miscarriages. The first group, the experimental one, included 30 women with a history of RM whose ages ranged from 28 to 35 and had had a history of abortion twice or more and no successful delivery. The members of this group also experienced abortion for unknown causes in the first trimester of their pregnancy. The second group, which was the control group, included 30 women who were under 35 with at least two children and had no history of miscarriages.

### 2.2. Sample Collection and Preparation

5 cc blood samples were collected from women in both groups and centrifuged at 1500 × *g* for 10 min at 4°C. Sera were separated and stored at -70°C [27].

### 2.3. ^1^H NMR Analysis

600 *μ*l of serum samples were mixed with 60 *μ*l of deuterium oxide (D_2_O) and transferred to 5 mm NMR tubes [29] and the CPMG spin echo method was carried out with ^1^H NMR spectroscopy (450 kHz) in the Central Laboratory of Isfahan University [30].

### 2.4. Chemometrics Analysis

NMR spectra were analyzed in MATLAB software using the ProMetab program. The peaks were normalized and divided into 1500 bins and chemical shifts converted into matrices in Excel for multivariate analysis using the statistical option of the MetaboAnalyst software. The spectra were normalized using mean centering and Pareto scaling and analyzed by PLS-DA to get the score plots and the loading plots [31].

### 2.5. Metabonomic Analysis

Chemical shifts of the differentiated metabolites were obtained and identified using the Human Metabolome Database [25] (HMDB) in both the experimental and control groups. The pathway analysis option of the Metaboanalyst.ca [26] website was used to determine the changed metabolic pathways.

## 3. Results

**Figure 1:**
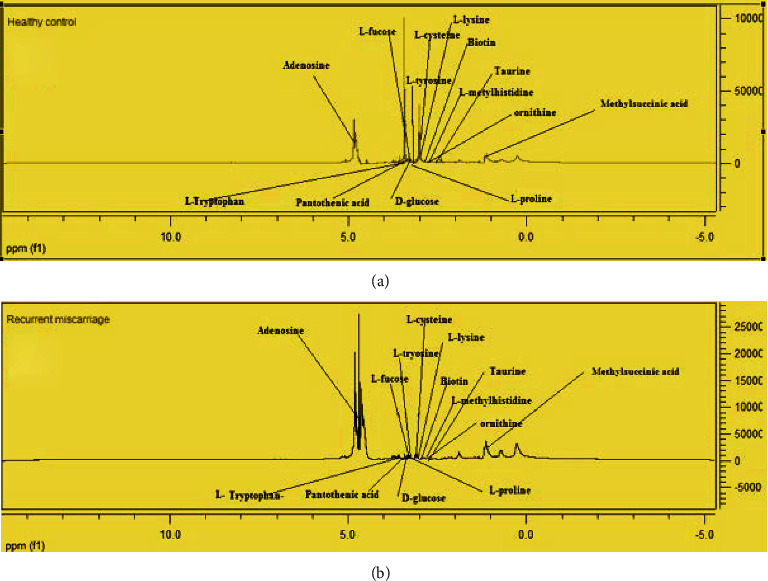
^1^H NMR spectra of women with recurrent miscarriages and healthy controls with identified differentiating metabolites.

**Figure 2:**
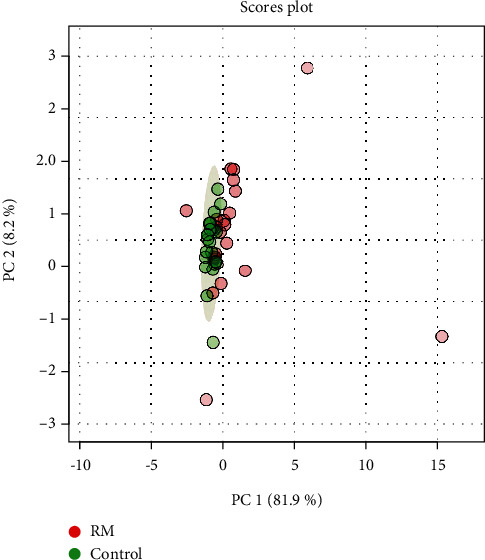
The 2D score plot of PLS-DA between selected components. RM: pink dot; control: green dot.

**Figure 3:**
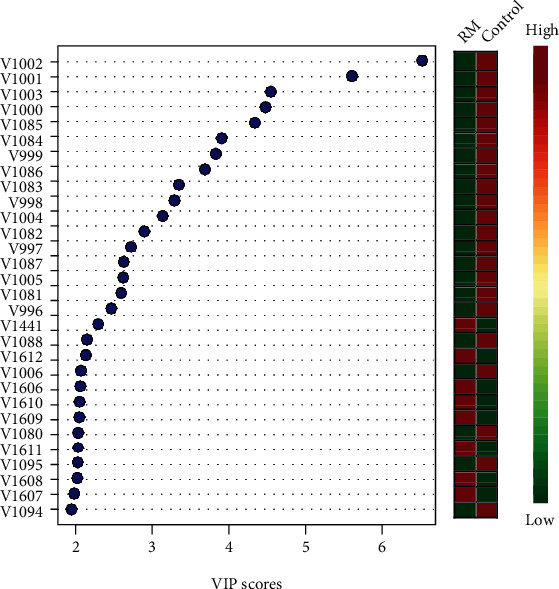
Permutation test for model variation demonstrating differentiating variables identified by PLS-DA. The higher the VIP scores, the more significant they are. The colored boxes on the right indicate the levels of the metabolites.

**Figure 4:**
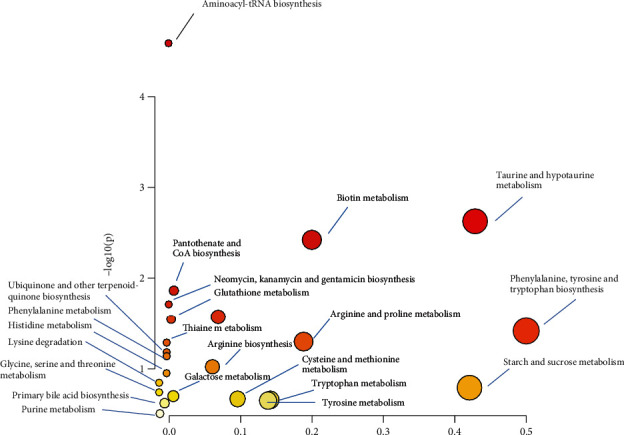
Pathway impact of metabolic pathways, the higher and darker the circles and the closer to the *y*-axis, the more significant and higher the impact.

(1) The typical serum ^1^H NMR spectra of women with RM and healthy controls are shown in Figure 1. The spectra contained very high-intensity signals from adenosine, D-glucose, L-tyrosine, pantothenic acid, L-cysteine, L-lysine, biotin, ornithine, and taurine in patients with RM (Figure 1(b)) in comparison to healthy controls (Figure 1(a)).

(2) The NMR spectra dataset was subjected to normalization by sum and data scaling by mean centering. PLS-DA was done to illustrate the differences in the metabolic profiles (Figure 2). The score plot revealed the distinct separation of the RM group from the control group.

(3) Variable Importance in Projection (VIP) scores indicate the numbers of variables representing the different chemical shifts which are the discerning metabolites (Figure 3).

(4) The chemical shifts corresponding to the list of variables were entered into HMDB, and differentiating metabolites were obtained and presented in Table 1 containing the increased D-glucose, methyl succinic acid, L-proline, and adenosine levels and decreased L-fucose, 1-methylhistidine, L-tyrosine, pantothenic acid, L-lysine, biotin, L-tryptophan, ornithine, L-cysteine, and taurine levels. The difference in metabolite levels of the individuals with and without RM is seen in Table 1.

(5) MetaboAnalyst 5.0 was used to perform a more detailed analysis of the most relevant RM pathways and networks. The pathway analysis is demonstrated in Figure 4 and presented in Table 2 showing that phenylalanine, tyrosine and tryptophan biosynthesis, taurine and hypotaurine metabolism, starch and sucrose metabolism, biotin metabolism, arginine and proline metabolism, tryptophan metabolism, tyrosine metabolism, cysteine and methionine metabolism, pantothenate and CoA biosynthesis, and also glutathione metabolism were altered in RM. Phenylalanine, tyrosine, and tryptophan biosynthesis (impact = 0.50) and taurine and hypotaurine metabolism (impact = 0.43) pathways were identified as potential target pathways for RM.

## 4. Discussion

The primary objective of this study was to investigate the sera of patients with RM with those who had no record of miscarriages to determine if there were any metabolic and pathway differences. The results of the study showed the existence of differentiating metabolites and pathways, which are presented in Tables 1 and 2, respectively.

An earlier study on sera of women with idiopathic recurrent abortions identified increased amounts of specific amino acids. It was also hypothesized that lysine, glutamine, threonine, L-arginine, histidine, phenylalanine, and tyrosine are altered metabolites involved in excessive inflammatory reactions and vascular dysfunction; also, they are related to poor endometrial receptivity [27]. In this study, L-tyrosine, L-lysine, L-cysteine, L-proline, and L-tryptophan have changed as well. Such changes in the amino acids mentioned above might have influenced inflammatory reactions and vascular dysfunction.

It was reported that taurine and hypotaurine help maintain redox homeostasis in gametes. Both taurine and hypotaurine neutralize lipid peroxidation products; hypotaurine further neutralizes hydroxyl radicals [32]. The taurine and hypotaurine metabolism altered in missed abortion in the early gestational period [26]. Taurine, which ensures normal mitochondrial and endoplasmic reticulum function, reduces the risk of apoptosis and premature death [33]. Since taurine decreased among the patients with RM, such reduction of taurine might lead to higher risks of mitochondrial dysfunction and apoptosis.

Biotin is necessary for maintaining the reproductive function, and some human fetal malformations may be caused by biotin deficiency [34]. Biotin is required to maintain normal pregnancy, fetal development, and reproductive performance during the late stage of gestation [35, 36]. Accordingly, the rate of biotin was lower among the patients with RM. This reduction indicates that one of the reasons for RM might be the reduction of biotin.

Indoleamine 2,3-dioxygenase (IDO), which is the tryptophan-degrading enzyme, inhibits the proliferation and activation of T cells [37]. IDO increases the tolerance of the immune system and maintains the embryo against an immune response [38]. It was reported that in comparison to normal pregnancy, the activity and expression of IDO were low in patients with unexplained RM [39]. Moreover, tryptophan metabolism and sphingolipid metabolism are important potential targets for miscarriage prevention [40]. In agreement with previous results in our study, it was revealed that tryptophan metabolism decreased among patients with RM. The reduction of L-tryptophan might influence tryptophan metabolism; therefore, such a decrease adds to a higher probability of RM.

The female reproductive system is influenced by thyroid hormones since these hormones regulate the functioning and development of uterine, placental tissues, and ovarian [41]. Besides, thyroid hormones are crucially important for pregnancy maintenance [42] and the development of the fetal brain [43].

Many reproductive disorders such as spontaneous abortion, infertility, and ovarian cysts are driven by hypothyroidism [41]. This study revealed that L-tyrosine, which is the precursor of thyroid hormones, decreased among women with RM. Therefore, the reduction of L-tyrosine might affect thyroid hormones, which might lead to a higher probability of RM.

Methionine transports methyl groups in methylation reactions such as DNA methylation, biological amines, and the synthesis of purines, proteins, and phospholipids during growth. One of the intermediate components of the methionine cycle is homocysteine which is involved in abortion. Hyperhomocysteinemia damages chronic vessels; as a result, it impairs the implementation of the fetus. Also, hyperhomocysteinemia by reducing the density of avascular villi causes spontaneous abortion. Furthermore, hyperhomocysteinemia determines coagulation dysfunction, which might lead to early pregnancy loss [43, 44]. In our study, cysteine and methionine metabolism pathway has changed among patients with RM. Such change in cysteine and methionine metabolism might result in coagulation dysfunction and RM.

Pantothenic acid (PA) is required to synthesize coenzyme A (CoA), which is vital for fatty acid metabolism. In general, CoA synthesizes and metabolizes carbohydrates, fat, and protein [45].

A metabolomics study on the amniotic fluid of spontaneous abortion showed that fatty acid and coenzyme A metabolism altered. Reduced biosynthesis of CoA could be related to observed differences in fatty acid metabolism [46]. A metabolomics study on determining metabolic alterations in pregnant dairy cows reported that the decreased maternal plasma PA was due to the transfer of PA to the fetus via the uterus [47]. In this study, the PA level of patients with RM decreased in comparison with the healthy group.

It is believed that pregnancy is a condition of increased oxidative stress due to impaired balance between prooxidants and antioxidants. The best antioxidants are glutathione and its related enzymes. There are reports that there might be a correlation between spontaneous abortions and low intracellular activity of glutathione peroxidase enzyme [48]. Numerous studies have highlighted that genetic polymorphisms which codify antioxidant enzymes are associated with an increased risk of oxidative stress-related diseases [49–51]. In another study, it was also found that the risk of RM correlates with glutathione transferase genes [51]. In this study, a change in the cycle of glutathione metabolism among the patients with RM was also observed. The change in this cycle might increase oxidative stress; therefore, there would be a higher probability of RM.

It was reported that not only thiamine deficiency increases stillbirths and spontaneous abortions but also it affects gestation outcomes and fetus viability [52]. Since thiamine deficiency might interfere with hormonal mechanisms, it might lead to some disorders such as unsuccessful fetus implantation, spontaneous abortion, and ovarian dysfunction [53]. Accordingly, it was revealed that thiamine metabolism as another metabolic cycle was also different among the patients with RM.

Earlier reports have demonstrated that increased fetal loss was observed at the extremes of glycemia in diabetic and nondiabetic pregnancy [54]. Another study revealed that early pregnancy loss increased with marked hyperglycemia in diabetic pregnancy [55]. As presented in the results, D-glucose was seen to increase among women with RM. Therefore, it may be concluded that such an increase might contribute to a higher risk of RM.

However, to detect the biomarkers and get a clearer picture of the metabolic profiles, the study should be carried out on a much larger number of samples and the metabolite concentrations identified with LC-MS.

## 5. Conclusion

In this study, ^1^H NMR was used to analyze the sera and metabolic profiles of patients with RM with those of healthy ones; 14 metabolites were detected and 2 potential target pathways, the taurine-hypotaurine metabolism and phenylalanine, tyrosine, and tryptophan biosynthesis, were deemed to be of prime importance. Therefore, the changed metabolites and metabolic pathways might widen the horizons for further studies to investigate the extent to which the increase or decrease of these metabolites might lead to the occurrence of RM. Further studies are needed with a larger number of samples to clearly identify the metabolite biomarkers using LC-MS.

## Figures and Tables

**Table 1:** The list of differentiating metabolites obtained by HMDB.

No.	Metabolite name	HMDB number	Levels	*p* values
1	L-Tyrosine	HMDB0000158	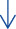	*p* < 0.02
2	L-Fucose	HMDB0000174	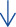	*p* < 0.01
3	Pantothenic acid	HMDB0000210	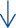	*p* < 0.02
4	Biotin	HMDBB0000030	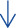	*p* < 0.02
5	Taurine	HMDB0000251	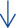	*p* ≤ 0.02
6	L-Lysine	HMDB0000182	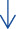	*p* ≤ 0.02
7	L-Cysteine	HMDB0000574	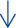	*p* ≤ 0.02
8	D-Glucose	HMDB0000122	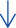	*p* < 0.02
9	L-Proline	HMDB000162	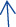	*p* < 0.02
10	Ornithine	HMDB0000214	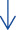	*p* < 0.02
11	L-Tryptophan	HMDB0000929	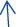	*p* < 0.02
12	Methyl succinic acid	HMDB0001844	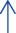	*p* < −0.04
13	Adenosine	HMDB0000050	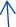	*p* < 0.02
14	1-Methylhistidine	HMDB0000001	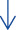	*p* < 0.02

**Table 2:** The associated metabolic pathways of each biomarker.

	Total	Expected	Hits	Raw *p*	−log_10_(*p*)	Impact
Aminoacyl-tRNA biosynthesis	48	0.46	5	5.49*E* − 05	4.26*E* + 00	0.00
Taurine and hypotaurine metabolism	8	0.08	2	2.37*E* − 03	2.63*E* + 00	0.43
Biotin metabolism	10	0.10	2	3.76*E* − 03	2.42*E* + 00	0.20
Pantothenate and CoA biosynthesis	19	0.18	2	1.36*E* − 02	1.87*E* + 00	0.01
Neomycin, kanamycin, and gentamicin biosynthesis	2	0.02	1	1.93*E* − 02	1.72*E* + 00	0.00
Glutathione metabolism	28	0.25	2	2.50*E* − 02	1.60*E* + 00	0.00
Galactose metabolism	27	0.26	1	2.67*E* − 02	1.57*E* + 00	0.07
Phenylalanine, tyrosine, and tryptophan biosynthesis	4	0.04	1	3.82*E* − 02	1.42*E* + 00	0.50
Arginine and proline metabolism	38	0.37	2	5.03*E* − 02	1.30*E* + 00	0.19
Thiamine metabolism	7	0.07	1	6.59*E* − 02	1.18*E* + 00	0.00
Ubiquinone and other terpenoid-quinone biosynthesis	9	0.09	1	8.40*E* − 02	1.08*E* + 00	0.00
Phenylalanine metabolism	10	0.10	1	9.29*E* − 02	1.03*E* + 00	0.00
Arginine biosynthesis	14	0.14	1	1.28*E* − 01	8.94*E* − 01	0.06
Histidine metabolism	16	0.15	1	1.45*E* − 01	8.39*E* − 01	0.00
Starch and sucrose metabolism	18	0.17	1	1.61*E* − 01	7.92*E* − 01	0.42
Lysine degradation	25	0.24	1	2.17*E* − 01	6.63*E* − 01	0.00
Glycine, serine, and threonine metabolism	33	0.32	1	2.77*E* − 01	5.58*E* − 01	0.00
Cysteine and methionine metabolism	33	0.32	1	2.77*E* − 01	5.58*E* − 01	0.10
Tryptophan metabolism	41	0.40	1	3.32*E* − 01	4.78*E* − 01	0.14
Tyrosine metabolism	42	0.41	1	3.39*E* − 01	4.70*E* − 01	0.14
Primary bile acid biosynthesis	46	0.45	1	3.65*E* − 01	4.38*E* − 01	0.01
Purine metabolism	65	0.63	1	4.76*E* − 01	3.23*E* − 01	0.00

## Data Availability

The data is available on request to the corresponding author.
